# Design, Modeling, and Evaluation of the Eddy Current Sensor Deeply Implanted in the Human Body

**DOI:** 10.3390/s18113888

**Published:** 2018-11-11

**Authors:** Rajas Prakash Khokle, Karu P. Esselle, Desmond J. Bokor

**Affiliations:** 1 School of Engineering, Macquarie University, Sydney 2109, NSW, Australia; Karu.esselle@mq.edu.au; 2 Faculty of Medicine and Health Sciences, Macquarie University, Sydney 2109, NSW, Australia; desbok@iinet.net.au

**Keywords:** implantable sensor, eddy current sensor, micromotion sensor, orthopedic implants, electromagnetic FEM analysis

## Abstract

Joint replacement surgeries have enabled motion for millions of people suffering from arthritis or grave injuries. However, over 10% of these surgeries are revision surgeries. We have first analyzed the data from the worldwide orthopedic registers and concluded that the micromotion of orthopedic implants is the major reason for revisions. Then, we propose the use of inductive eddy current sensors for in vivo micromotion detection of the order of tens of μm. To design and evaluate its characteristics, we have developed efficient strategies for the accurate numerical simulation of eddy current sensors implanted in the human body. We present the response of the eddy current sensor as a function of its frequency and position based on the robust curve fit analysis. Sensitivity and Sensitivity Range parameters are defined for the present context and are evaluated. The proposed sensors are fabricated and tested in the bovine leg.

## 1. Introduction

Human locomotion largely depends on the health of the knee joint. The knee joint consists of the lower end of the femur (thigh bone), the upper end of tibia (shin bone) and patella (knee cap). They are protected by different cartilages, ligaments and tendons. Various thigh and calf muscles attached to these bones provide the necessary strength and enable motion. To reduce the friction and enable smooth operation, these bones are covered by a thin synovial membrane that provides lubrication. In a healthy knee, all these parts work in perfect harmony. However, with age, this process starts failing, and a person’s movement is restricted. Alternatively, if a person has a disease like arthritis or suffers from injuries and accidents involving the knee, it results in reduced mobility and pain. Some of the common features of a damaged knee joint are bone spurs and narrow joint spaces with damaged ligaments and cartilage loss. When a disease or injury is in an advanced state, it is not possible to cure it using any medicine or physiotherapy. In such cases, knee replacement or knee arthroplasty is the only solution. The knee replacement surgery or the Total Knee Arthroplasty /Replacement (TKA or TKR) aim to resurface the damaged knee parts to improve the joint condition that reduces the pain and increases the mobility of the person. Similar procedures exist for hip, shoulder, ankle, wrist and elbow joints.

However, these surgeries often require a revision. The ratio of primary surgeries to revision surgeries is known as revision burden. Worldwide, over sixteen major countries maintain some form of the orthopedic register. Out of these, Australia [1,2], New Zealand [3], the United Kingdom [4], Portugal [5] and the Netherlands [6] keep an extensive record of all kinds of the surgeries. Table 1 shows the revision burden for different arthroplastic surgeries. Apart from these, the USA [7], Switzerland [8], and Belgium [9] report a revision burden of 8.7%, 9.3% and 7.5% for TKA, respectively. Some of these registers, especially from Australia, the UK, and New Zealand, also make note of reasons for revision. The reasons for the revision are loosening (28.7%), pain (20.9%), infection (22.4%), instability (6.3%), implant erosion (5.4%), arthrofibrosis (3.8%), fracture (2.6%), misalignment (2.3%) and others (7.6%). To understand these reasons for revision, the process of osseointegration, implant stability and micro motion are discussed next.

### 1.1. Osseointegration, Implant Stability, and Micromotion

Osseointegration is the term used to denote an intimate contact between the bone and the metallic implants. The principles and process of osseointegration are well known and well understood in the literature [10,11,12,13]. After the surgery, the newly formed tissues start occupying the voids and spaces of the micro-porous surface of the implant. The cuts and drills in the tibial or femoral bones are repaired by hematoma formation and mesenchymal tissue. These are later replaced by the woven bone tissues. This is followed by the lamellar bone remodelling and the growth of the bone marrow.

There is evidence that the high relative motion between the implant and the host bone leads to the ingrowth of the fibrous tissues rather than bone [14]. Thus, the success of good fixation of the implant by osseointegration depends on a stable interface between the implant surface and the bone [15,16,17]. In [18], a study of the stability of femoral components having a non-porous distal stem was done in the canine model. It was found that the relative micromotion was 34 μm just after the surgery, which is decreased to 5 μm within a year that indicated extensive bone regrowth and its excellent stability. It was concluded that proximally porous uncemented femoral components are crucial in providing the initial stability to the implant. This research led to the development of the porous implants. Furthermore, in [19], a computational model was developed that explored the implant design features that resisted loosening. The finite element analysis of the contact mechanics of the press-fit hip implants also showed that minimizing the micromotion, especially during the post-operative situation, should provide an adequate stability for the implant to promote the osseointegration [20].

In [21], it was found that the bone ingrowth was inhibited by the intermittent micromotion of the implants in rabbits. About 20 cycles of 0.5 mm motion were applied once a day. The motion induces shear strain that causes fibrous tissue formation instead of the bone. In [22], osseointegration under the bio-mechanical stress–strain and the bone formation and resorption was studied in the beagle dogs. Their investigations showed that the micromotion of less than 30 μm at the bone implant interface does not interfere with the bone growth. In [23], local micromotion measurement on the femoral stem for Total Hip Replacement (THR) is reported. The failure of the cement-less THR is mainly attributed to the aseptic loosening and stability of the implants [24,25]. About 150 μm of micromotion has been considered as the limit for avoiding the fibrous tissue formation and promoting the bone ingrowth [23,25]. In [26], micromotion generation as an effect of applied stress and torque on the bone (dental implants) of different densities was studied. It was found that a mean force of 62.7 N shows the maximum displacement of 71.9 μm.

Thus, the micromotion of the implant, especially during the early stages of recovery, makes osseointegration difficult and gives rise to the implant instability. This, in due course of time, creates a painful situation for the patient and later warrants a re-surgery.

### 1.2. Modalities to Measure the Micromotion

In [27], various techniques currently employed to detect the micromotion of the implant are discussed. They are classified into two groups: imaging based and implant integrated sensor based. Imaging techniques include radiography [28], computed tomography [29], Magnetic Resonance Imaging [29,30], and FDG-PET (Fluorodeoxyglucose-Positron Emission Tomography) [31]. Implant integrated sensors involve the use of accelerometers, vibrometry and differential variable reluctance transducer (DVRT).

While radiography is a 2D assessment of a 3D process, computed tomography offers better quantification of osteolysis. MRI is more useful for the evaluation of the periprosthetic tissue (as MRI provides an excellent soft tissue contrast). FDG-PET is quite cost intensive and provides accuracy only when combined with other modalities. A detailed procedure to obtain the micromotion information in the lab settings is described in [32]. It utilizes X-ray opaque markers made from tantalum, inserted at specific locations in the bone followed by the X-ray imaging at regular intervals. Recently, in [33], a new technique based on micro computed tomography (micro-CT) and radiopaque markers is proposed to provide a full field micromotion measurement at the entire bone–implant interface. This study has been performed for the cement-less femoral stem implants in a human cadaver. It had a standard deviation of 4 μm and accuracy of over 20 μm.

Implant integrated sensors that use accelerometers with vibrometry are described in [34]. Well fixed implants show linear behavior, while loosened ones show an acoustic behavior. However, the specificity and sensitivity of vibrometry is estimated to be only 20% higher than the radio graphs. Another contact based system based on the differential variable reluctance transducer (DVRT) has been developed in [35]. In this setup, the commercially available DVRT sensors from Microstrain Inc. (Williston, MA, USA) were used in contact-based configuration. These sensors also had to be calibrated frequently to account for the frequency dependent hysteresis. Moreover, the electronics for motion detection was outside the body.

However, these systems cannot be used for a prolonged monitoring of the orthopedic implant. It is not possible to utilize the X-ray techniques as it would mean a visit by the patient to a radiologist everyday. In addition, the daily radiation dose from these methods would be detrimental to the patient’s health. The contact based configuration is not suitable for an implantable micromotion sensor as it would mean changing the design of an orthopedic implant, which has been optimized through years of research.

Therefore, an implanted sensor that can sense the micromotion of an orthopedic implant remotely (without modifying or touching the implant) can be very useful in reducing the revision surgeries. The holes drilled for positioning the drilling jig on the bone while doing the surgery are left after doing the procedure. Holes have a diameter of about 3 mm and length of 12 to 15 mm. These holes are quite good for implanting the proposed micromotion sensor. These holes are drilled at a distance ranging from about 3 mm to 15 mm from the surface of the tibial/femoral implant. Therefore, the proposed remote micromotion should have a stand-off range from about 3 mm to 15 mm and resolution of about 10 s of micrometers. Keeping in view the non-magnetic and lossy dielectric nature of the human tissue, we propose using inductive eddy current (EC) sensors for this purpose [36]. In [37], authors have discussed how eddy current sensors can be utilized for motion detection. In this article, we critically analyze the problem of micromotion detection of the orthopedic implant and discuss the simulation strategy, experimental methods and obtained results.

**Figure 1:**
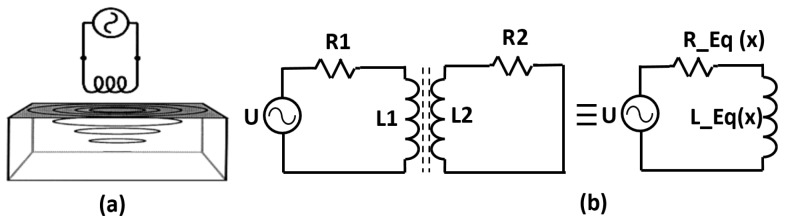
(**a**) physical model and (**b**) equivalent circuit of the Eddy Current sensor.

For the displacement measurement, the physical model and an equivalent circuit model of an eddy current loop is shown in Figure 1. The alternating current in the primary coil generates the alternating magnetic field. This induces eddy currents in the metallic target such that the secondary magnetic field opposes the primary magnetic field. The coil and the target are considered as weakly coupled primary coil and shorted secondary coil, respectively. This makes the equivalent circuit model shown in Figure 1b. The primary coil has its own resistance, whereas for induced eddy currents that constitute secondary coil in the equivalent circuit, the power dissipation gives rise to its own secondary resistance. If the target moves away from the coil, the amount of eddy current induced decreases therefore the resistance reflected to the primary coil decreases while the inductance increases. This impedance change is utilized for the displacement measurement. The equivalent inductance, resistance and Q factor of the this simple model can be given by the Equations (1)–(3) [38,39]:

(1) $$L_{eq}=L_{1}-\frac{\omega^{2}M^{2}\left(x\right)L_{2}}{R_{2}^{2}+\omega^{2}L_{2}^{2}},$$

(2) $$R_{eq}=R_{1}+\frac{\omega^{2}M^{2}\left(x\right)R_{2}}{R_{2}^{2}+\omega^{2}L_{2}^{2}},$$

(3) $$Q_{eq}=\frac{\omega L}{R}=\frac{\omega \left(L_{1}-\frac{\omega^{2}M^{2}\left(x\right)L_{2}}{R_{2}^{2}+\omega^{2}L_{2}^{2}}\right)}{R_{1}+\frac{\omega^{2}M^{2}\left(x\right)R_{2}}{R_{2}^{2}+\omega^{2}L_{2}^{2}}}.$$

Here, R1 and L1 are the resistance and self-inductance of the sensor coil depending on the material and structure of the coil; R2 and L2 are the equivalent resistance and self-inductance of the target depending on the eddy current path, conductivity σ and relative permeability $\mu_{r}$ of the target; and ω is the exciting angular frequency of the power source, and M is the mutual inductance between the sensor coil and the target that depends on the relative position *x* between the sensor and target.

However, these expressions do not account for the dispersive and inhomogeneous nature of the human body. In addition, as the angular frequency of operation (ω) increases, the electric field strength also increases as per Maxwell’s equation. In addition, with frequency, the current along the loop length varies that further adds to the electric fields. The human body therefore affects the operation of such a sensor both by absorbing the power and affecting the sensor characteristics. Furthermore, the loop is printed on a dielectric substrate and has a rectangular cross section which may not be negligible with respect to the shorter dimension of the loop. Thus, various analytical methods available in literature [40,41] are not suitable for investigating the response of an eddy current sensor implanted inside the human body. Therefore, full wave analysis by numerical methods is frequently employed [42,43,44,45].

Implanting the sensor inside the human bone at the research stage is both a complicated and expensive proposition. Furthermore, experimental evaluation alone cannot provide answers to the questions like the effect of power dissipation and the electromagnetic field distribution in the human tissue, so we setup numerical simulations. Then, we perform analysis in the free space as well as in the human body with respect to the frequency of operation and stand-off distance. The frequency at which the sensor works inside the body without any appreciable change is found out through these simulations. Then, the experimental setup is done and the free space results at different stand-off distances and frequencies are verified. Finally, the experiments with the bovine femur are conducted and the sensor characteristics are evaluated.

## 2. Methods

### 2.1. Numerical Modeling of Sensors

**Figure 2:**
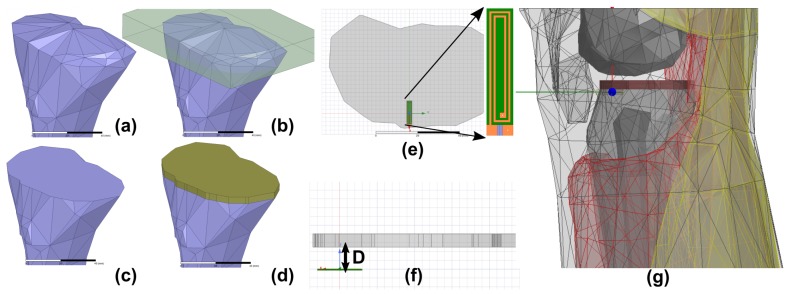
(**a**) model of human tibia from Ansys male body model; (**b**) a cuboid with 5 mm thickness intersecting with bone; (**c**) resurfaced tibial bone obtained by subtracting the cuboid from tibia; (**d**) placing the tibial orthopedic implant on top of the resurfaced plate; (**e**) top view showing the eddy current loop with respect to the orthopedic implant. (inset: zoomed-in view of the eddy current loop); (**f**) side view showing the distance (D) between the plate and the eddy current loop; (**g**) complete leg model constructed around the eddy current sensor.

Ansys High Frequency Structure Simulator (HFSS) that uses a Finite Element Method to solve Maxwell’s Equations numerically is exclusively used in this study for the numerical modeling of the implantable eddy current sensor. First, a two-turn loop of length 10 mm and width 2 mm with the line width of 0.2 mm on Rogers 6010 substrate is designed in the XY plane. Then, the tibial bone from Ansys Human body model is imported as shown in Figure 2a. A rectangular box is defined on the top side of this tibia (Figure 2b). On boolean subtraction, a flat surface on top of the bone is obtained (Figure 2c). This is equivalent to resurfacing the tibial bone using a power saw during the real TKR procedure. This surface is thickened to form a thick sheet that has the shape of an orthopedic tibial implant and Titanium material is assigned to it (Figure 2d). This is followed by drilling cylindrical holes of 3 mm diameter and 15 mm depth in the bone below the orthopedic plate. Then, the eddy current sensor loop is inserted below the tibial implant. The orientation of the sensor and the implant is shown in Figure 2e–g. Finally, various muscle tissues from the human body model are imported and the complete leg is constructed.

**Figure 3:**
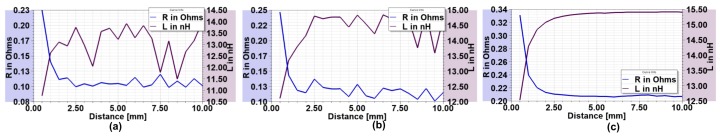
(**a**) results from the default solver settings; (**b**) the effect of setting tighter convergence criteria; (**c**) the effect of setting tighter convergence criteria along with smaller mesh size for substrate and second order basis functions.

Setting up the correct simulation for the proposed eddy current sensor is a non-trivial task as the default solver settings do not produce physically correct results (Figure 3a). Since the model will be simulated at several frequency points at different stand-off distances, it is necessary to ensure that the simulation produces a converged and physically correct solution with a minimum amount of CPU and RAM consumption. The most important simulation settings that affect the accuracy of simulation are convergence criteria, meshing, and the order of the basis functions used. These settings are evaluated against the memory required, the CPU time and the convergence of the results. To get the physically viable and correct results, the sensor position is varied along the *Z*-direction and the impedance characteristics of the loop are evaluated. The tibial implant is displaced from 0.5 mm to 10 mm in steps of 0.5 mm and resistance and inductance are evaluated at 100 MHz.

An eddy current sensor is not used beyond its self resonant frequency (SRF), so we define solution frequency as SRF of loop. For determining the SRF, the loop is initially simulated with solution frequency of 5.35 GHz, which corresponds to the free space half wavelength perimeter of the loop (56 mm). With this initial simulation, SRF of the loop is found to be 3.3 GHz. For all further simulations, this frequency is used as the solution frequency. Using a smaller solution frequency results in 20% less simulation time and 46% less RAM.

It is found that reducing the default convergence criteria to $|\Delta S_{max}|<0.001$ and ∠s<0.1 gives slightly smoother curves (Figure 3b). In addition, further tightening of convergence criteria does not improve the result despite the increase in the solution time. Hence, order of basis function and meshing are considered for further improvement. The default setup converges with a lower number of mesh elements on the substrate. Consequently, the fields are interpolated at many positions. The interpolation error gives rise to the mesh noise which is found to be more than the reflected field due to eddy currents, especially at high stand-off distance. Therefore, to ensure that the field at the point of interest is calculated by the solver directly rather than interpolation, and the maximum length of any side of tetrahedron on the substrate is limited to 0.5 mm. This setting also has the advantage of reaching convergence in a low number of passes as the initial mesh is much denser than the automatic mesh generated by the solver.

Figure 3c shows the smooth variation in resistance and reactance when the second order basis functions are used along with the substrate meshing with the maximum tetrahedron length of 0.5 mm and the convergence criteria as $|\Delta S_{max}|<0.001$ and ∠ΔS<0.1. From Table 2, it can be seen that the use of second order basis functions has a clear advantage in terms of number of passes (50% less), number of mesh elements (63% less) and CPU time (37%) required to reach the convergence criteria. However, it consumes 47% more RAM and so the actual simulations are run on a dual socket server machine with 128 GB RAM.

### 2.2. Experimental Setup

**Figure 4:**
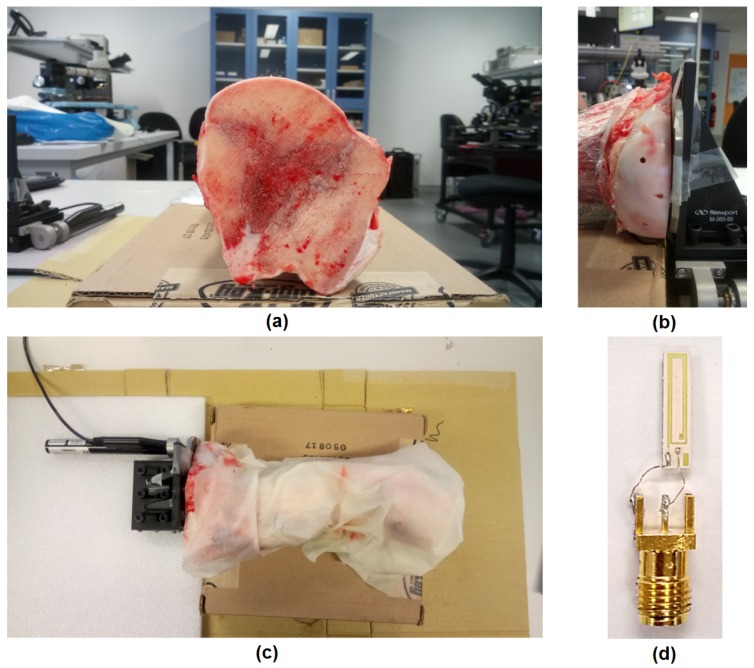
Cow bone preparation for experimental validation. (**a**) bone cleaved to create a flat surface; (**b**) placement of bone and titanium implant with the position of three holes (Φ=3 mm) drilled at 3 distances—5 mm, 10 mm and 15 mm; (**c**) bone covered with the wet towel to avoid dessication; (**d**) two-turn fabricated inductive sensor connected with an SMA connector with 0.1 mm wires.

The eddy current sensor is fabricated on Rogers 6010 substrate using traditional PCB manufacturing setup by Lintek Pty. Ltd. at Quenbeyan in New South Wales, Australia. The copper layers are silver-gold immersion coated for preventing the oxidation. The sensor pads were connected to the SMA connector using 0.1 mm diameter copper wires of length 10 mm (Figure 4d).

A bovine femur bone bought from the butcher shop is used for studying the effect of the bone and muscle tissue. One end of this bone is cleaved to get a flat surface (Figure 4a). Then, the tissue surrounding the bone are removed and the bone surface is revealed. Then three holes of diameter 3.5 mm are drilled at a distance of 5 mm, 10 mm and 15 mm from the edge of the flat end with a depth of about 20 mm by using a power drill. The holes are drilled in different lines to avoid the effect of bone–air discontinuity. The bone is aligned in such a way that the titanium implant mounted on the micromotion stage can touch the flat surface of the bone (see Figure 4b). The entire bone is covered with a wet towel to avoid the dessication of the bone and the muscle tissues during measurements. It is shown in Figure 4c.

**Figure 5:**
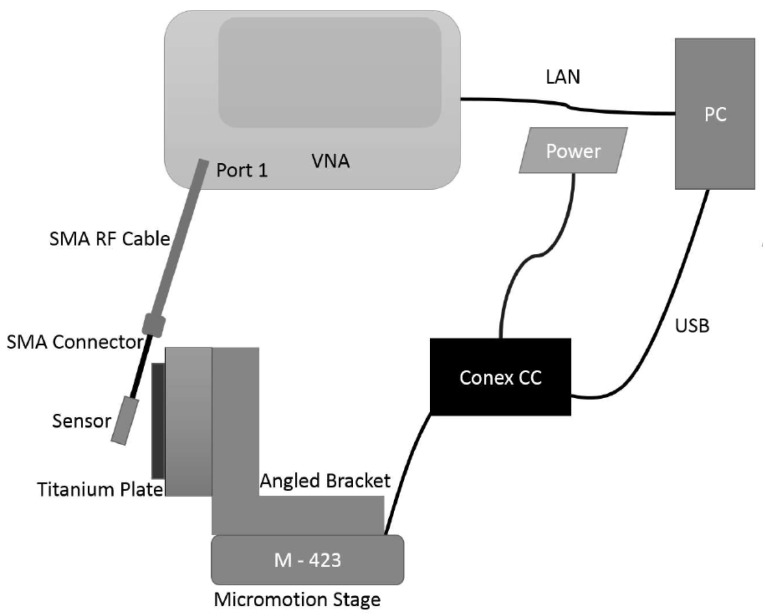
Schematics of the entire experimental setup in free space showing the micromotion stage, micromotion controller, VNA and PC.

To generate the motion of the order of tens of micrometers, a motorized stage is constructed using a Newport M423 Linear translation stage (Irvine, CA, USA) and a Newport right angled bracket. The stage is driven by Newport TRB25CC actuator integrated with a CONEX CC controller (Irvine, CA, USA) and Conex power supply. The controller is connected to the PC to enable the programming of the motion. To automate the entire measurement process, Agilent PNA-X (Santa Clara, CA, USA) is connected to the PC via LAN cable. The schematic of the entire setup is shown in the Figure 5.

First, the connections to the Conex CC controller via a COM port and to the VNA (Agilent PNA-X N5242A) via LAN port are initialized. Then, the controller state is checked and it is brought to a zero position by issuing a homing command. This is followed by the calibration of the VNA. The VNA is calibrated by using the open, short and broadband matched loads (Agilent 85052B 3.5 mm cal kit). The calibration is quite important in these experiments as the loop that has very low impedance, especially at a lower frequency, is directly connected to the 50 Ω SMA connector of VNA. This introduces the mismatch of the order of tens of dBs. Hence, it is necessary to have a good calibration standard in place. However, since the calibration is done until the co-axial cable end point and not the SMA connector with wires, their effect is de-embedded by using a two port fixture removal tool. The two port results are exported in S2P format. This file is imported into VNA in fixture correction tool. This de-embeds the effect of the SMA connector and the 10 mm length wires.

Once the calibration is completed, the target is moved to the end position such that the sensor touches the target. This is taken as zero position. Then, the motion stage is moved by the predetermined amount and its position is read back and verified. Then, the VNA is triggered with proper settings for the frequency range, the number of frequency points, if bandwidth (set to 100 Hz) and the number of averaging points (20 points per sample). The point-to-point averaging method is selected. Low IF bandwidth, high averaging and proper calibration and embedding ensure good quality of the measurements.

After the measurement is complete, the data is read in a binary block. The results are S-parameters ($S_{11}$) in dB magnitude and phase form, from which they are converted into impedance (*Z*) with real and imaginary parts to extract resistance (R) and inductance (L) of the loop using Equation (4). The motion stage is again activated to go to the next position and the entire ‘move–measure–acquire’ loop is executed until the motion stage reaches the maximum displacement position:

(4) $$Z_{11}=\left(\frac{S_{11}+1}{S_{11}-1}\right)50\;R=ℜe\left(Z_{11}\right)\;L=\frac{ℑm(Z_{11})}{2\pi F}.$$

## 3. Results and Analysis

### 3.1. Numerical and Experimental Results in Free Space

A MATLAB code is written that implements the algorithm mentioned in Section 2.2. The results are captured at every 10 MHz increment from 10 MHz to 1.1 GHz. Initially, the sensor is tested in free space. This is followed by testing in the cow bone at three different distances 5 mm, 10 mm and 15 mm.

**Figure 6:**
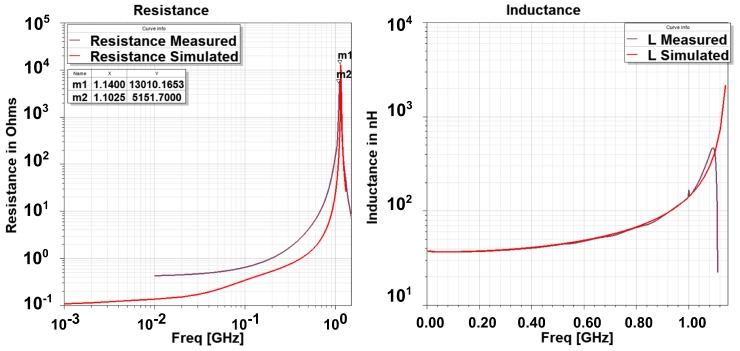
Comparison of simulated and measured resistance and inductance as a function of frequency.

The measured and simulated resistance and inductance are compared in Figure 6. The measured resistance is higher than the simulated resistance. This may be due to the wire resistance or SMA connector loss that is not accurately modeled by the simulation but dominates the small loop resistance at lower frequency, which is less than 0.5 Ω. Overall, all of the experimental curves show a behavior similar to that of simulations, especially inductance and the self resonant frequency.

**Figure 7:**
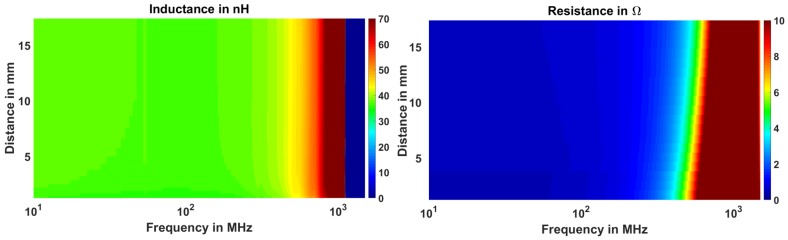
Measured inductance and resistance as a function of stand-off distance and frequency.

The impedance parameters not only change with the stand-off distance, but they also change with the frequency as shown in Figure 7. At higher frequencies, the resistance shows higher change in the resistance with displacement. However, this figure does not give any quantitative measure of the sensitivity or possible range of the sensor. For this purpose, we perform a curve fit analysis and define the appropriate sensitivity and range parameters (discussed in Section 3.3).

### 3.2. Experiments in a Cow Bone

**Figure 8:**
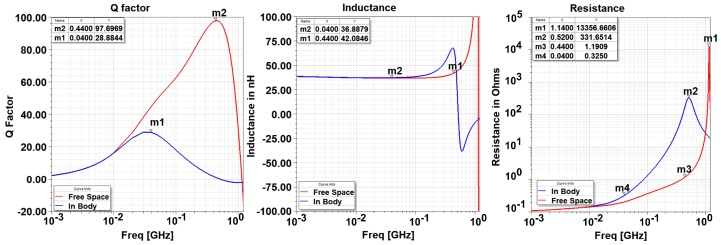
Comparison of the free space and in body impedance properties of the two-turn eddy current sensor.

The femur bone from a cow is prepared as explained in the previous sections. The stand-off distance has a tolerance of ±0.25 mm due to the human errors. The sensor is inserted in these holes and the motion in 10 μm steps is provided by the micromotion stage. Figure 8 shows the effect of the bone on the impedance characteristics of the EC sensor. Compared to the free space, the self resonant frequency of the sensor decreases from 1.14 GHz to 0.52 GHz. Furthermore, since the resistance increases, the peak Q factor decreases from 97.7 to 28.8. In addition, the frequency at which the Q factor peaks decreases from 0.44 GHz to 0.04 GHz.

**Figure 9:**
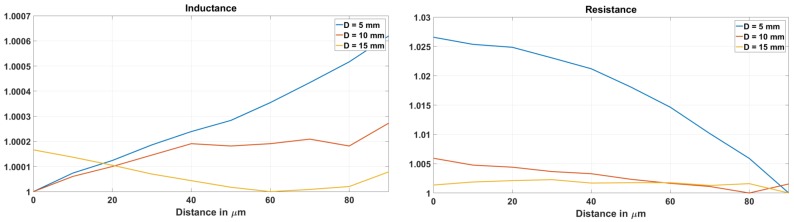
Change in inductance, resistance and Q Factor with 10 μm motion of the implant at stand off distance of 5 mm, 10 mm and 15 mm.

The impedance parameters of the loop as a function of the displacement of the target at 40 MHz frequency are plotted in Figure 9. Resistance sensitivity is higher than the inductance sensitivity. With an increase in the stand off distance, the sensitivities for both the resistance and inductance decrease. However, the decrease in the inductance sensitivity is less than the decrease in the resistance sensitivity.

### 3.3. Evaluation of the Sensitivity of EC Sensor

The inductance (L), resistance (R), and the Q-factor changes nonlinearly with the stand-off distance (see Figure 3c). The dependence of R, L and Q factor parameters on distance can be curve fitted using Equation (5), where *y* can be resistance, inductance or Q factor, *x* is the stand-off distance and *a*, *b*, and *c* are co-efficients of the curve fit:

(5) $$y=ax^{b}+c.$$

**Figure 10:**
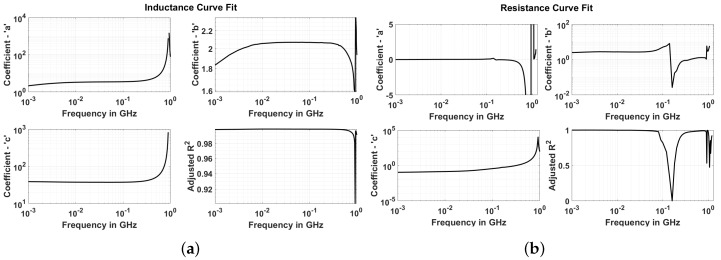
Curve fit coefficients for inductance (**a**) and resistance (**b**).

For inductance and Q factor, *c* will be a positive value, whereas *a* and *b* will be negative. For resistance, *a* and *c* will be positive, whereas *b* will be negative. This is taken into account while writing a program for curve fit. Thus, for inductance, the equation used is $y=c-ax^{-b}$ and, for resistance, the equation used is $y=ax^{-b}+c$. Consequently, all of the coefficients are calculated positive by curve fit algorithm. This facilitates log–log plot of the values. The result of the curve fit for inductance, resistance and Q factor are shown in Figure 10. The following observations can be made from the curve fit analysis.

1. The curve fit at maximum frequency points is exceptionally good as seen from adjusted $R^{2}$ graph where its value is near to 1.
2. At $F_{SRF}$, the adjusted $R^{2}$ drops to a low value for the three parameters. This indicates a failed curve fit. Correspondingly, the curve fit coefficients have discontinuity or abrupt change at $F_{SRF}$ and so are not reliable indicators of the actual behavior. This behavior is expected as the resistance becomes infinite and the reactance abruptly changes to negative value; therefore, there are no current flows through the loop and thus the curve fit fails.
3. The adjusted $R^{2}$ drops at a certain frequency before SRF for resistance (and so the Q factor). It is around 160 MHz. This also indicates that curve fit does not reproduce the data points well. In addition, it can be noted that at this frequency ($F_{Worst}$), co-efficient *a* becomes negative for resistance. Co-efficient *c* always remains positive which merely indicates that the resistance is positive.

**Figure 11:**
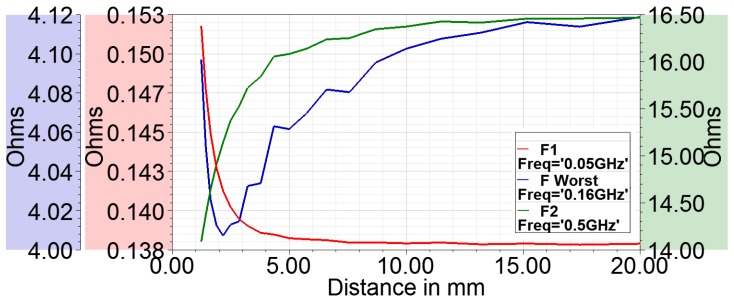
Resistance–distance curves at three frequencies $F_{1}<F_{Worst}$, $F_{Worst}$ and $F_{2}>F_{Worst}$ showing the reversal of trend in eddy current sensor at $F_{Worst}$.

**Figure 12:**
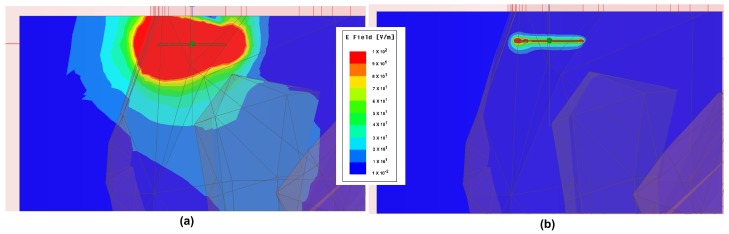
Distribution of E fields in body at (**a**) 1 GHz; (**b**) 30 MHz.

To investigate this issue of bad curve fit around $F_{Worst}=160$ MHz, the resistance–distance curves are plotted for three different frequencies, at $f_{1}<F_{Worst}$, $F_{Worst}$ and $f_{2}>F_{Worst}$ in Figure 11. At lower frequency $f_{1}$, the resistance decreases with increasing distance. At $f_{2}$, the resistance increases with distance. $F_{Worst}$ is the transition frequency. Around this frequency, the resistance change is not well behaved. Therefore, the curve fit of the power law form fails at this frequency. Consequently, operating the sensor around this frequency is not recommended. At higher frequencies, the sensor may be operated and possibly with higher sensitivity but also with higher losses in body as well as loop. To investigate this further, the electric field distribution is shown in Figure 12. It can be seen that, at higher frequency, E fields are much stronger and have a wide distribution in the bone and muscle tissue, whereas, at 30 MHz, it is negligible and localized close to the sensor. Therefore, from the point of view of power dissipation in human body, it is better to operate around 20–30 MHz region.

### 3.4. Definition of EC Sensor Parameters

The curve fit coefficients do not yield any substantial information by themselves. However, they can be used to calculate the output (R, L or Q factor) at any distance. This is used to define useful sensor parameters. First, the sensitivity is defined as the change in the output quantity *y* (inductance, resistance, Q Factor) for the 10 μm displacement of the target expressed in logarithmic scale (to the base 10) by Equation (6):

(6) $$S_{10\mu m}\left(\mathrm{dB}\right)=10log\left(\frac{\Delta y}{y}\right).$$

The logarithmic scale is used to handle very small numbers and produce meaningful graphs. According to this definition, sensitivity of 10 dB means 1 part in 10 is changed for 10 μm motion, 20 dB corresponds to 1/100, 30 dB to 1/1000 and so on.

**Figure 13:**
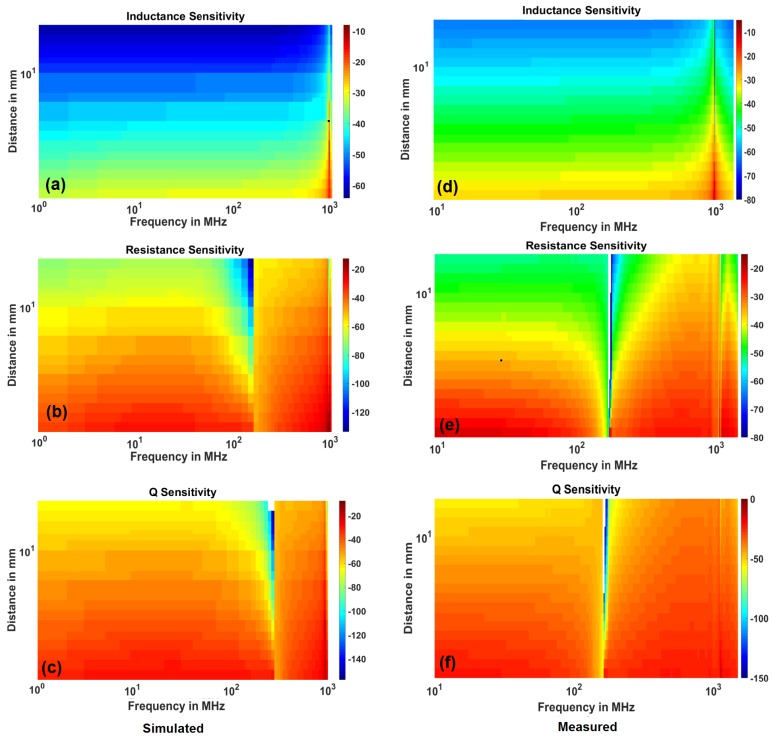
Comparison of simulated and measured sensitivities for inductance, resistance and Q factor.

Using Equation (6), the sensitivity of the sensor with respect to inductance, resistance and Q factor is calculated at different frequencies between 1 MHz and $F_{SRF}$ and stand-off distances between 1 mm to 20 mm. To facilitate the comparison, the simulated and measured results of the sensitivity analysis is plotted in Figure 13. The colors represent the sensitivity in the dB value. The sensitivity decreases with the stand-off distance and increases with the frequency as expected. Especially, inductance sensitivity shows this behavior strikingly well. The resistance (and Q factor) sensitivity shows a drop at $F_{W}orst$ frequency as explained above. However, due to this phenomenon, there is an optimal frequency of operation between 1 MHz and $F_{Worst}$ where sensitivity peaks (at 20 MHz).

**Figure 14:**
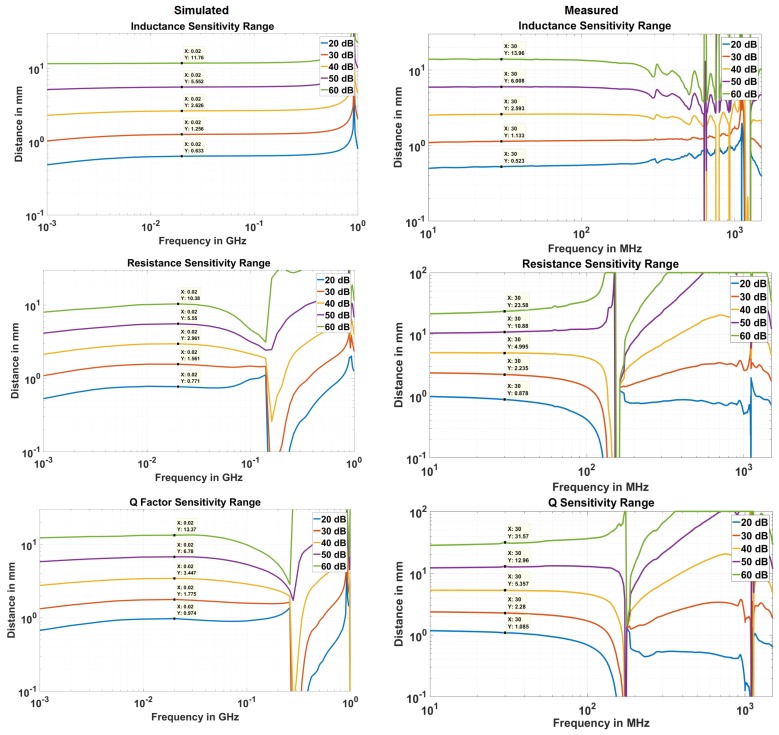
Comparison of simulated and measured sensitivity ranges for inductance, resistance and Q-factor for 10 μm displacement.

Based on the definition in Equation (6), sensitivity range is defined at *x* dB. This is the range at which the sensor can detect 10 μm displacement if the detection circuit can produce measurable output for *x* dB change. If the sensor is placed beyond this range, it cannot be used to detect the 10 μm motion. Therefore, the maximum range that a sensor can have for a given level of sensitivity, called sensitivity range, is plotted in Figure 14.

The simulated and measured results match very closely. However, the measured results show more sensitivity especially at higher distances than the simulated case. This may be due to the extra length of the rectangular loop created by the wires connecting SMA to the sensor head. Although de-embedding adjusts inductance and resistance and fixes the SRF of the sensor, it cannot prevent the EM fields generated by the extra wires. This issue can be rectified once the complete integrated circuit for measurement is developed. The agreement of the results, especially the $F_{Best}$ and $F_{Worst}$ frequencies, validate the numerical simulations.

It should be noted that Q factor is the quantity derived mathematically from resistance and inductance measurements. Therefore, if the inductance and resistance could not be measured at a given distance, the Q factor cannot be calculated as well. However, if they can be measured, then Q factor can give higher resolution or sensitivity. The increased Q factor sensitivity range, therefore, is merely representative of increases sensitivity and not the actual distance at which the device can work.

## 4. Future Directions

The work presented in this article confirms the feasibility of utilizing an eddy current sensor for detecting the micromotion of the orthopedic implant. Future research involves determining the bio compatible encapsulation for the sensor. Since the bio-compatible material will be in close proximity of the sensor, its effect on the Electromagnetic field distribution must be investigated thoroughly. Furthermore, its effect on the sensitivity and range of the sensor will have to be evaluated. To provide the energy to the sensor and for communication with the external interrogator, a miniaturized bone implantable antenna has to be designed and tested [46].

## 5. Conclusions

A numerical model of an eddy current sensor implanted in the tibial bone of the human knee is created. Then, the reliable and correct simulation settings were determined and validated by experiments. It is seen that implanting the sensor inside the bone decreases the self resonant frequency of the sensor as well as sensitivity. A new definition of sensitivity is proposed, based on the curve fit analysis, that measures the amount of impedance change with stand-off distance and frequency for 10 μm change in the position of the orthopaedic implant. It is found that the frequencies in the range of 10 MHz to 40 MHz are most suitable considering maximum sensitivity and low power dissipation in the surrounding tissue. The maximum possible stand-off distance for a given sensitivity level is also calculated. These results are expected to be useful for an integrated circuit designer who can design a low power circuit for creating a completely standalone implantable sensor. The proposed sensor is designed, fabricated and tested in a bovine leg to demonstrate the feasibility of the design.

## Figures and Tables

**Table 1:** Number of orthopedic surgeries from the registers of different countries and revision burden in % for the different joints.

	Australia	United Kingdom	Holland	New Zealand	Portugal
Hip Primary	440,841	800,683	125,391	110,208	4384
Hip Revision	57,819	89,023	16,991	16,251	648
**Burden %**	**11.59**	**10.00**	**11.93**	**12.85**	**12.87**
Knee Primary	544,075	875,585	116,780	95,821	4110
Knee Revision	48,502	54,278	10,360	6739	291
**Burden %**	**8.18**	**5.83**	**8.14**	**6.57**	**6.61**
Shoulder Primary	29,068	17,300	2077	7305	111
Shoulder Revision	3338	2045	203	571	9
**Burden %**	**10.30**	**10.57**	**8.90**	**7.24**	**7.5**
Ankle Primary	1662	3185	122	1261	17
Ankle Revision	376	358	15	179	1
**Burden %**	**18.44**	**10.10**	**10.94**	**12.43**	**5.55**
Elbow Primary	2738	1639	107	476	66
Elbow Revision	536	507	38	81	4
**Burden %**	**16.37**	**23.62**	**26.20**	**14.54**	**5.71**

**Table 2:** Effect of order of basis function on the system resources. $N_{p}$—number of passes to reach convergence criteria.

Order	$N_{p}$	Mesh Elements	RAM	CPU Time (Min:S)
First Order	8	76719	4.81 GB	56:31
Second Order	4	28400	7.08 GB	35:52

## Abbreviations

The following abbreviations are used in this manuscript:

TKA	Total Knee Arthroplasty
TKR	Total Knee Replacement
EC	Eddy Current
